# Effect of dietary salt intake on epithelial Na^+^ channels (ENaC) in vasopressin magnocellular neurosecretory neurons in the rat supraoptic nucleus

**DOI:** 10.1113/JP274856

**Published:** 2017-07-30

**Authors:** Kaustubh Sharma, Masudul Haque, Richard Guidry, Yoichi Ueta, Ryoichi Teruyama

**Affiliations:** ^1^ Department of Biological Sciences Louisiana State University Baton Rouge LA 70803 USA; ^2^ Department of Physiology University of Occupational and Environmental Health Kitakyushu 807‐8555 Japan

**Keywords:** electrophysiology, neurohypophysis, oxytocin

## Abstract

**Key points:**

- A growing body of evidence suggests that epithelial Na^+^ channels (ENaCs) in the brain play a significant role in the regulation of blood pressure; however, the brain structures that mediate the effect are not well understood.
- Because vasopressin (VP) neurons play a pivotal role in coordinating neuroendocrine and autonomic responses to maintain cardiovascular homeostasis, a basic understanding of the regulation and activity of ENaC in VP neurons is of great interest.
- We show that high dietary salt intake caused an increase in the expression and activity of ENaC which resulted in the steady state depolarization of VP neurons.
- The results help us understand one of the mechanisms underlying how dietary salt intake affects the activity of VP neurons via ENaC activity.

**Abstract:**

All three epithelial Na^+^ channel (ENaC) subunits (α, β and γ) are located in vasopressin (VP) magnocellular neurons in the hypothalamic supraoptic (SON) and paraventricular nuclei. Our previous study demonstrated that ENaC mediates a Na^+^ leak current that affects the steady state membrane potential in VP neurons. In the present study, we evaluated the effect of dietary salt intake on ENaC regulation and activity in VP neurons. High dietary salt intake for 7 days caused an increase in expression of β‐ and γENaC subunits in the SON and the translocation of αENaC immunoreactivity towards the plasma membrane. Patch clamp experiments on hypothalamic slices showed that the mean amplitude of the putative ENaC currents was significantly greater in VP neurons from animals that were fed a high salt diet compared with controls. The enhanced ENaC current contributed to the more depolarized basal membrane potential observed in VP neurons in the high salt diet group. These findings indicate that high dietary NaCl intake enhances the expression and activity of ENaCs, which augments synaptic drive by depolarizing the basal membrane potential close to the action potential threshold during hormonal demand. However, ENaCs appear to have only a minor role in the regulation of the firing activity of VP neurons in the absence of synaptic inputs as neither the mean intraburst frequency, burst duration, nor interspike interval variability of phasic bursting activity was affected. Moreover, ENaC activity did not affect the initiation, sustention, or termination of the phasic bursting generated in an intrinsic manner without synaptic inputs.

## Key points

- A growing body of evidence suggests that epithelial Na^+^ channels (ENaCs) in the brain play a significant role in the regulation of blood pressure; however, the brain structures that mediate the effect are not well understood.
- Because vasopressin (VP) neurons play a pivotal role in coordinating neuroendocrine and autonomic responses to maintain cardiovascular homeostasis, a basic understanding of the regulation and activity of ENaC in VP neurons is of great interest.
- We show that high dietary salt intake caused an increase in the expression and activity of ENaC which resulted in the steady state depolarization of VP neurons.
- The results help us understand one of the mechanisms underlying how dietary salt intake affects the activity of VP neurons via ENaC activity.

**Abbreviations**
ENaC: epithelial Na^+^ channel
PVN: paraventricular nucleus
SON: supraoptic nucleus
VP: vasopressin

## Introduction

Vasopressin (VP) is a neurohypophysial hormone synthesized by magnocellular neurosecretory cells in both the supraoptic (SON) and paraventricular (PVN) nuclei in the hypothalamus. The release of VP into the general circulation from the neurohypophysis is enhanced in response to hyperosmolarity (Brimble & Dyball, 1977), hypovolaemia (Harris *et al*. 1975) and hypotension (Khanna *et al*. 1994) to produce pressor and antidiuretic effects (Antunes‐Rodrigues *et al*. 2004). In addition to the release of VP from the neurohypophysis, VP is also released from the soma and dendrites of magnocellular neurons in the PVN to increase the activity of the parvocellular neurons within the PVN that project to the rostral ventrolateral medulla (Son *et al*. 2013). This action, in turn, affects sympathetic outflow and blood pressure (Dampney, 1994). VP thus appears to play a pivotal role in coordinating neuroendocrine and autonomic responses to maintain cardiovascular homeostasis.

The non‐voltage dependent, amiloride sensitive, epithelial Na^+^ channel (ENaC) is expressed in the apical membrane of epithelia involved in trans‐epithelial Na^+^ transport (Canessa *et al*. 1994; Harris *et al*. 2008), such as in the renal collecting duct, distal colon and respiratory epithelium (Kellenberger & Schild, 2002). ENaCs were also found in several regions of the brain involved in cardiovascular control, such as the PVN, SON and choroid plexus (Amin *et al*. 2005). Several studies demonstrated that central administration of amiloride, or its analogue benzamil attenuated sympathetic activity and hypertension in several animal models of hypertension (Gomez‐Sanchez & Gomez‐Sanchez, 1994, 1995; Nishimura *et al*. 1998, 1999; Keep *et al*. 1999; Wang & Leenen, 2002; Wang *et al*. 2003), indicating that ENaCs in the brain play a significant role in the regulation of blood pressure. Our previous study demonstrated that all ENaC subunits (α, β and γ) are specifically expressed in VP neurons in the SON and PVN, and that ENaCs affect the membrane potential of the neurons by mediating a Na^+^ leak current (Teruyama *et al*. 2012). Because the release of VP is largely regulated by the pattern and frequency of action potentials of the synthesizing neurons (Poulain & Wakerley, 1982), these findings suggest that modulation of the firing activity by ENaCs plays a critical role in the regulation of hormone release from VP neurons. However, the regulation of ENaC activity in VP neurons remains largely unknown.

Dietary salt intake is known to influence ENaC activity in aldosterone sensitive epithelia (Garty & Palmer, 1997). For instance, dietary salt deficiency caused a marked increase in the abundance and activity of ENaCs in the renal collecting duct (Masilamani *et al*. 1999) and taste cells (Lin *et al*. 1999) in rats to promote Na^+^ retention and appetite, respectively. However, the effect of dietary salt intake on the activity of ENaC in VP neurons was not examined. The present study was conducted to elucidate whether the expression and activity of ENaCs in VP neurons are affected by dietary salt intake. The findings from this study provide some insight on how ENaCs contribute to the changes in the activity of VP neurons in response to high dietary salt intake.

## Methods

### Ethical approval

All protocols for animal experiments were approved by the Institutional Animal Care and Use Committees of Louisiana State University. Male wild type Wistar rats were obtained from Envigo (Indianapolis, IN, USA).

### Animals and diets

Transgenic Wistar rats that express VP‐eGFP fusion protein in VP neurons (Ueta *et al*. 2005) were originally provided by Dr Ueta of the University of Occupational and Environmental Health in Japan, and a colony was established in facility at Louisiana State University. For real‐time RT‐PCR and immunocytochemical experiments, wild type Wistar rats were fed a NaCl deficient, control, or high salt diet *ad libitum* for 10 days. For electrophysiological experiments, the VP‐eGFP transgenic Wistar rats were fed a control or high salt diet *ad libitum* for 10–14 days. The NaCl adjusted diet, NaCl deficient (0.01–0.02% NaCl; TD.90228), control (0.4% NaCl) and high NaCl diet (8% NaCl; TD.92012) were purchased from Teklad diet (Indianapolis, IN, USA). All rats weighed between 260–300 g and were housed on a 12:12 h light–dark cycle and were provided water *ad libitum*.

### Real‐time RT‐PCR

Animals were deeply anaesthetized with ketamine‐xylazine (9:1; 100 mg kg^−1^ i.p.) and perfused through the heart with 0.01 m sodium phosphate buffered saline (PBS; pH 7.2) for immunocytochemistry or with cold artificial cerebral spinal fluid (ACSF) in which NaCl was replaced by equiosmolar sucrose for real‐time RT‐PCR and electrophysiology.The brains were removed quickly following the transcardial perfusion and sectioned in ice‐cold ACSF in the coronal plane at a thickness of 500 μm by a vibrating microtome (Leica VT1200, Mannheim, Germany). The tissues containing the entire SON were collected using a punch‐tool (inner diameter 1.0 mm) and saved in RNA*later* (Qiagen, Valencia, CA, USA). One of the kidneys from each animal was also removed, minced, and saved in RNA*later*. The tissue samples were homogenized in TriReagent (Sigma‐Aldrich, St Louis, MO, USA) using a tissue lyser (Qiagen). The lysate was then treated with chloroform (Thermo Fisher Scientific) and centrifuged at 12,000 *g* for 15 min. Total RNA was precipitated from the aqueous stage using isopropyl alcohol, and then washed with ethanol. The precipitate was suspended in 20 μl RNAase‐free water. Isolated mRNA was reverse transcribed to cDNA using oligo dT and M‐MLV reverse transcriptase (Sigma‐Aldrich) and used in real‐time RT‐PCR analysis. The ABI ViiA‐7 sequence detection system (ABI Applied Biosystem, Grand Island, NY, USA) was used in conjunction with SYBR Select Master Mix (ABI Applied Biosystem). The following primer sets were used for αENaC (forward: 5′‐CCCAAGGGAGTTGAGTTCTG‐3′; reverse: 5′‐AGGCGCCCTGCAGTTTAT‐3′), βENaC (forward: 5′‐GGACCAGAGCTAAATATCACC‐3′; reverse: 5′‐CGGTAGTTGAACTCTTGGAAGTAGA‐3′), γENaC (forward: 5′‐CCAGTACAGCCAGCCTCTG‐3′; reverse: 5′‐CTGGTACAACTGGTAGTAGCAATACAT‐3′), and cyclophilin B (forward: 5′‐CTTGGTGTTCTCCACCTTCC‐3′; reverse 5′‐ACGTGGTTTTCGGCAAAGT‐3′). All primer sequences were blasted on the National Center for Biotechnology Information database to determine specificity of the targeted gene. All samples were measured in triplicate and the two closest cycle threshold (Ct) values were averaged for each sample. The average Ct value of the housekeeping (cyclophilin B) gene was subtracted from that of the target gene to obtain the ΔCt value. There were no differences in the Ct values for the cyclophilin B among groups. The ΔCt values of the designated control group were averaged and subtracted from each sample to generate the ΔΔCt value. Relative change was then calculated for each sample by using the formula 2^−ΔΔCt^. All results are shown as relative changes in comparison to the controls. Outliers were detected by the robust non‐linear regression method and were excluded from the analysis.

### Immunocytochemistry

Animals were transcardially perfused with 4% paraformaldehyde in 0.1 m sodium phosphate buffer (PB; pH 7.2) following PBS and decapitated. The heads were postfixed in the same fixative for 1–3 days. The brains were extracted and infiltrated with 20% sucrose in 0.1 m PB for cryoprotection overnight. Coronal sections were transected at 40 μm by a sliding microtome (Leica SM2010R; Mannheim, Germany). The free‐floating brain slices were incubated with the primary antibodies against αENaC, βENaC, or γENaC at dilutions of 1:2000, 1:500 and 1:4000, respectively, in PBS containing 0.5% Triton X‐100 (PBST) for 48–72 h with continuous gentle agitation at 4°C. The α‐, β‐ and γENaC subunit antibodies were raised in rabbit and were a kind gift from Dr Mark A. Knepper (National Institutes of Health (NIH), Bethesda, MD, USA). The production and characterization of these ENaC subunit antibodies has been previously described in great detail (Masilamani *et al*. 1999).

For light microscopy, the brain sections were subsequently incubated with biotinylated goat anti‐rabbit IgG (1:200 in PBST; Vector, Burlingame, CA, USA) for 4 h, followed by the ABC complex (Vector) for 1 h. Finally, the sections were incubated with diaminobenzidine (DAB; Vector) for 6 min to visualize the immunoreactivity. The brain sections were mounted on gelatin‐coated slides, dehydrated, cleared, and cover slipped with Permount (Thermo Fisher Scientific). Light microscopic images were acquired digitally (Eclipse 80i equipped with a digital camera, DS‐QiMc, Nikon, Tokyo, Japan). Digital images were minimally altered in ImageJ software (NIH) with changes in dynamic range.

For fluorescent confocal microscopy, the brain slices were incubated with a secondary antibody (goat anti‐rabbit) conjugated with DyLight 594 (Jackson ImmunoResearch, West Grove, PA, USA) for 4 h at room temperature. The brain slices were examined and confocal images (1024 × 1024; 0.232 μm pixel^−1^) were acquired with a confocal microscope (Leica TCS SP2 spectral confocal microscope) using a 63× oil immersion objective (n.a. = 1.4). The optical section thickness was 1 μm. To obtain the line plot profile of the optical density of immunoreactivity within neurons, a 10‐pixel (2.32 μm) width line (plot reference line) was drawn using ImageJ software (NIH) from the outline of the neuron to the nucleus on the long axis. A plot reference line was drawn in a neuron that was optically sectioned at the centre of its cell body in *Z*‐stacked confocal images. Neurons that had the outline‐to‐nucleus distance of less than 10 μm were not included. A plot of optical density (OD) over the distance from the beginning of the plot reference line was obtained using the Plot Profile function of ImageJ. The location of the peak, decay constant and amplitude of each line profile was obtained using AxoGraph X.

### Electrophysiology

#### Slice preparation

The brains were removed quickly following the transcardial perfusion and coronal slices (250 μm) containing the SON were collected using a vibrating microtome (Leica VT1200). The brain slices were kept in ACSF (in mm: 125 NaCl, 2.5 KCl, 1 MgSO_4_, 1.25 NaH_2_PO_4_, 26 NaHCO_3_, 20 d‐glucose, 2 CaCl_2_, 0.4 ascorbic acid, pH of 7.3–7.4, with an osmolality of 290–300 mosmol kg^−1^ H_2_O) that also included 2 μm benzamil to prevent an intracellular Na^+^‐dependent ‘run‐down’ observed in the cells expressing ENaC (Garty & Palmer, 1997; Kellenberger *et al*. 1998; Staub *et al*. 2000). The benzamil was then washed out only after establishment of a stable patch clamp.

#### Whole cell patch clamp recording

Whole cell currents and membrane potentials were recorded digitally at 20 kHz and filtered at 5 kHz with a Digidata 1440A and an Axopatch 700B (Molecular Devices, Foster City, CA, USA) amplifier in conjunction with PClamp 10 software (Molecular Devices) on a Windows platform PC. VP neurons in the SON were visually identified with a microscope (Olympus BX50WI, Tokyo, Japan) equipped with a 40× water immersion lens (0.8 n.a.) under fluorescence illumination for detection of eGFP using a CCD camera. Recordings were taken using borosilicate electrodes (4–8 MΩ resistance) produced with a horizontal electrode puller (Model P‐1000 Micropipette puller, Sutter Instruments, Novato, CA, USA). The patch solution contained (in mm): 140 potassium gluconate, 1 MgCl_2_, 10 Hepes, 10 CaCl_2_, 2 MgATP, and 0.4 NaGTP, and 11 EGTA. The patch solutions also contained 0.2% biocytin (Sigma) to fill the patched cell (Teruyama & Armstrong, 2005, 2007) to confirm the cell type. The medium was saturated with 95% O_2_–5% CO_2_ and was warmed to 33–34°C during the recordings. Picrotoxin and DNQX (100 and 10 μm, respectively) were also added to ACSF to suppress the synaptic activity.

#### Data analysis

Phasic bursts were identified by the criteria used in previously published studies of VP neuron firing activity (Poulain *et al*. 1988; Sabatier *et al*. 2004; Li *et al*. 2007): at least one burst and one silent period must be observed within 5 min; the minimum burst and silence duration must be 3 s, and the minimum mean intraburst firing rate must be 2 spikes s^−1^. The interspike interval (ISI) was obtained from the time between subsequent action potential peaks. Interspike variability was quantified in the coefficient of variation (CV) which is the standard deviation of the ISI histogram divided by its mean. The ISI CV is useful for determining changes in spike train variability independently of changes in the mean firing rate (Koch, 1999). Averaged data are presented as the means + SEM, where *n* is the number of cells.

#### Specificity of benzamil

Amiloride and its analogue benzamil are known inhibitors of the degenerin/epithelial sodium channel superfamily of ion channels that includes both ENaC and the acid sensitive ion channel (ASIC). Although ASICs were also found in magnocellular neurons in the SON, the concentration of amiloride required to inactivate the ASICs in these neurons was high (100 μm), and 1 μm amiloride had little effect on ASIC activity (Ohbuchi *et al*. 2010). Amiloride and benzamil are also known inhibitors of the Na^+^–H^+^ exchanger (NHE) and the Na^+^–Ca^2+^ exchanger (NCX) (Kleyman & Cragoe, 1988). Amiloride at low doses is, however, reasonably specific for ENaCs as compared to the NHE and NCX (Kleyman & Cragoe, 1988). Furthermore, benzamil is nine‐fold more potent toward ENaCs with markedly lower relative potency (0.08‐fold) towards the NHE (Cuthbert & Fanelli, 1978). Therefore, the concentration of benzamil (1–2 μm) used in the present study is well below the IC_50_ of benzamil for ASIC, NHE and NCX.

### Statistics

Real‐time RT‐PCR data were analysed with ANOVA for comparison between treatment groups (control, NaCl deficient, and high NaCl diet). For electrophysiological data, the Student's *t* test was used for comparison between treatment groups (control vs high NaCl diet) and matched pairs analysis was used to test the effect of benzamil. Differences were considered to be statistically significant at *P* < 0.05. Box‐and‐whisker plots were used to represent numerical data: the mean and median are represented by an open circle and a line, respectively; the box extends to the quartiles of data points; the whiskers extend to the farthest data points; the outlier is represented by a filled circle.

## Results

### Changes in ENaC subunit mRNAs in response to dietary salt intake

**Figure 1:**
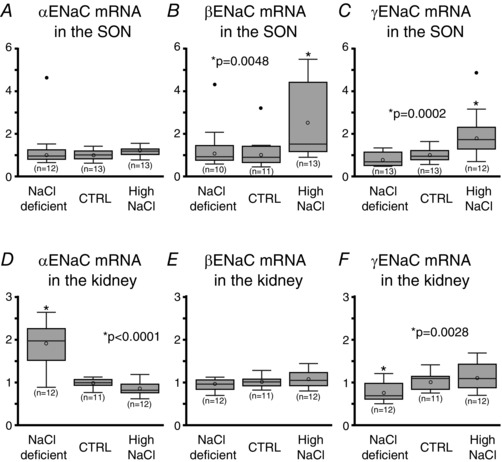
Effect of dietary salt intake on the expression of ENaC subunits in the SON and kidney *A*, high dietary salt intake for 7 days did not cause changes in the relative amount of αENaC mRNA in the SON. *B* and *C*, feeding a high NaCl diet for 7 days caused a significant increase in β‐ and γENaC mRNA in the SON, whereas the NaCl deficient and control diets did not cause changes in the abundance of β‐ and γENaC mRNA. *D*, feeding a NaCl deficient diet for 7 days caused a significant increase in αENaC mRNA abundance in the kidney. *E*, the abundance of βENaC mRNA was not affected by the dietary salt intake in the kidney. *F*, feeding a NaCl deficient for 7 days caused a significant decrease in γENaC mRNA in the kidney.

Thirty‐nine rats were fed either a NaCl deficient (*n = *13), control (*n = *13), or high NaCl (*n = *13) diet for 7 days and the relative amount of ENaC mRNAs in the SON were measured. No difference in the mean relative amount of αENaC mRNA was found among the experimental groups (Fig. 1 *A*). The mean relative amount of β‐ and γENaC mRNA in the SON was significantly higher in the high NaCl diet group than in the NaCl deficient and control diet groups (Fig. 1 *B* and *C*, respectively). In contrast to the SON, the mean relative amount of αENaC mRNA in the kidney was significantly higher in the NaCl deficient group compared to the control and high NaCl diet groups (Fig. 1 *D*). No difference in the mean relative amount of βENaC mRNA was found in the kidney among experimental groups (Fig. 1 *E*); however, the mean relative amount of γENaC mRNA was significantly lower in the NaCl deficient group compared to the control diet and high NaCl groups (Fig. 1 *F*).

### Changes in subcellular distribution of ENaC subunit immunoreactivity in response to high dietary salt intake

**Figure 2:**
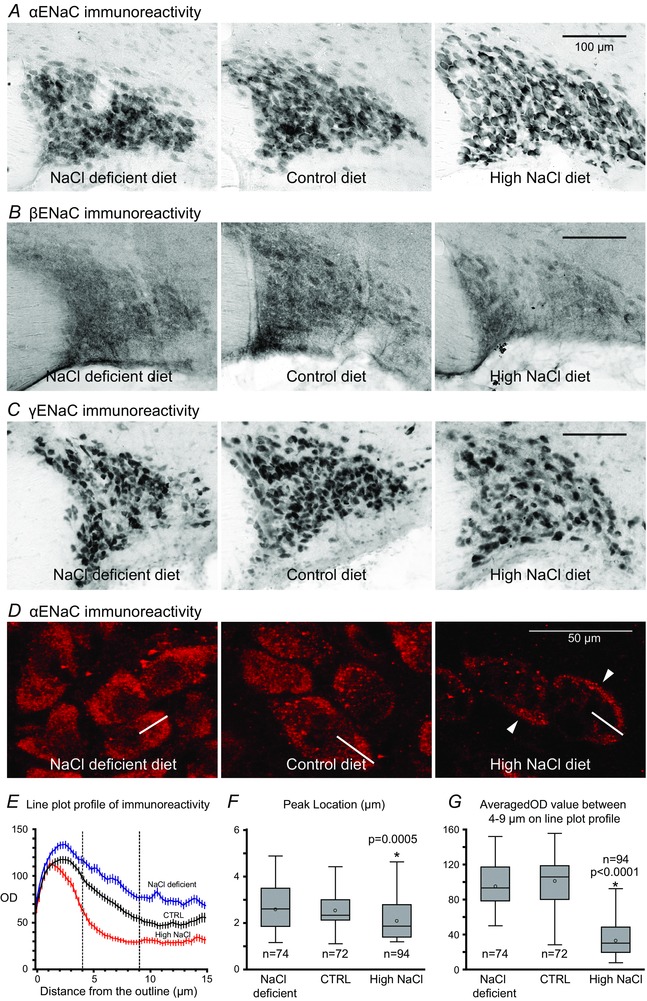
Photomicrographs showing immunoreactivity to αENaC (*A*), βENaC (*B*), and γENaC (*C*) in the SON from rats that were fed NaCl deficient, control, or a high NaCl diet *A*, strong αENaC immunoreactivity was found in MNCs regardless of dietary NaCl intake level; however, the immunoreactivity in MNCs from the NaCl deficient and control diet groups was diffused overall, whereas that in MNCs from the high NaCl group was sharper and outlined the soma more clearly. The difference in overall appearance of immunoreactivity appears to come from difference in the subcellular distribution of the immunoreactivity. *B*, only moderate and dispersed βENaC immunoreactivity was found in MNCs in the SON. There was no apparent difference in intensity or appearance of βENaC immunoreactivity in the SON among different dietary salt intakes. *C*, robust immunoreactivity was found in a limited number of MNCs in which immunoreactive product completely filled the soma regardless of the dietary NaCl. There were also many weakly labelled MNCs among strongly labelled MNCs. *D*, confocal microscopy of αENaC immunoreactivities in MNCs in the SON from rats fed NaCl deficient, control, and high NaCl diet. The αENaC immunolabelling in MNCs of NaCl deficient and control groups are mainly dispersed in the cytoplasm. Labelling in MNCs from the high NaCl diet group is concentrated towards the plasma membrane (arrows). 1 μm optical section. The white line in each image represents an example of the plot reference line where the profile of the optical density of immunoreactivity over the distance from the beginning (the outline of neuron) of the line was obtained. *E*, the averaged plot line profile of the optical density (OD) of immunoreactivity from the NaCl deficient (blue), control (black), and high NaCl diet (red) groups. Error bars represent SEM. *F*, the location of the peak OD value on the line plot profile. The peak OD value occurred significantly closer to the outline of each neuron from the high NaCl group than to those from the NaCl deficient or control groups. *G*, averaged OD values between 4 and 9 μm (indicated by vertical dashed lines in *E*) on the line plots profile. The mean averaged OD value of the high NaCl group was significantly lower than that of either the NaCl deficient or control groups.

Immunoreactivity of all ENaC subunits was found in magnocellular neurons in the SON (Fig. 2 *A*–*C*); however, the appearance of the immunoreactivity for each ENaC subunit was considerably different. Immunoreactivity of αENaC was reasonably intense and observed in most of the magnocellular neurons in the SON (Fig. 2 *A*). Immunoreactivity of βENaC was consistently moderate and diffused in appearance, and only a limited number of magnocellular neurons was delineated (Fig. 2 *B*). Some neurons were filled with extremely intense γENaC immunoreactivity along with neurons filled with somewhat weaker immunoreactivity (Fig. 2 *C*). There was no immediate noticeable difference in the overall intensity of αENaC immunoreactivity among the groups; however, the immunoreactive neurons were markedly well delineated in the SON from the high NaCl diet group compared to those of the NaCl deficient and control diet groups (Fig. 2 *A*). In contrast, no noticeable difference in overall intensity or appearance of β‐ and γENaC immunoreactivity was found among the experimental groups.

Subsequently, the subcellular distribution of ENaC subunits immunoreactivity was observed at a 1 μm optical dissection using a fluorescence confocal microscope. In all five rats examined, the αENaC labelling in neurons from the high NaCl diet group was concentrated towards the plasma membrane, while that from the NaCl deficient and control groups were mainly dispersed in the cytoplasm, with no distinct labelling at the plasma membrane (Fig. 2 *D*). The observations were remarkably consistent as similar subcellular distribution patterns were observed in all five animals within the same group. To quantify the subcellular distribution of αENaC immunoreactivity, plots of optical density (OD) of immunoreactivity over the distance from the outline of each neuron to the centre of its cell nucleus were obtained from 14 to 20 neurons from each animal (5 animals per group). The plots showed that the OD value for most of the neurons analysed increased sharply and peaked within 1.5–3.5 μm from the outline, then decreased gradually thereafter within the cytoplasm (Fig. 2 *E*). The plots from each group demonstrated that the mean peak OD value occurred significantly (Fig. 2 *F*; *P* = 0.0005) closer to the outline of the neurons (presumably near the cell membrane) in the high NaCl diet group (2.12 + 0.09 μm; *n* = 94) than in either the control group (2.52 + 0.75 μm; *n* = 72) or the NaCl deficient group (2.63 + 0.11 μm; *n* = 74). Moreover, the mean OD value of the distance between 4 and 9 μm on the plot reference lines (presumably in the cytoplasm 4–9 μm from the cell membrane) was significantly less (Fig. 2 *G*; *P *< 0.0001) in the high NaCl diet group (35.5 + 2.2; *n* = 94) than in the NaCl deficient group (96.6 + 3.0; *n* = 74) or controls (101.3 + 3.2; *n* = 72). These results confirmed that the αENaC labelling in neurons from the high NaCl diet group was concentrated towards the plasma membrane. Immuno‐fluorescence activity of βENaC was too weak for adequate observation at the subcellular level. The subcellular distribution of γENaC immuno‐fluorescence activity in most of the immunoreactive neurons was also not assessed, because a strong signal completely filled the entire cell body.

### Changes in benzamil sensitive current in response to high dietary salt intake

**Figure 3:**
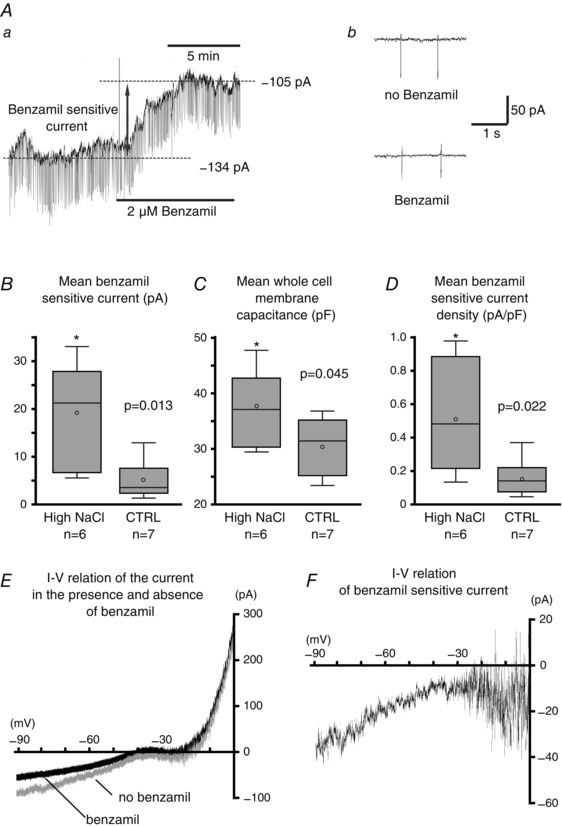
Effect of dietary NaCl on ENaC current *Aa*, an example of the effect of benzamil on the steady state current in a VP neuron. The steady state current was measured in voltage clamp while the cell was held at −80 mV. *Ab*, brief hyperpolarizing pulses (15 mV, 200 ms) were injected every 5 s to monitor the input resistance of the cell. Bath application of benzamil (2 μm) reduced the resting inward current and decreased conductance by approximately 1.5‐fold. A benzamil sensitive current was obtained by changes in the steady state current in response to its application. *B*, the mean benzamil sensitive current was significantly greater in VP neurons from rats that were fed a high NaCl diet in comparison to those fed a control diet (CTRL). *C*, whole cell membrane capacitance was higher in VP neurons from rats fed a high NaCl diet compared to those fed a control diet. *D*, the mean benzamil sensitive current density was significantly greater in VP neurons from rats that were fed a high NaCl diet than from those fed a control diet. The box plots in *B*–*D* represent the subpopulation of VP neurons that responded to the application of benzamil. *E*, the current–voltage (*I*–*V*) relation obtained by voltage ramp method (−90 to 0 mV in 5 s) in the presence of benzamil (black) and in the absence of benzamil (grey). *F*, The *I*–*V* relation of the benzamil sensitive current obtained by mathematical subtraction of the current in the absence of benzamil from that in the presence of benzamil.

Of 51 recorded VP neurons from 38 rats, 29 neurons were from rats (*n = *21) that were fed a high NaCl diet, and 23 neurons were from rats (*n = *17) that were fed a control diet. From these recorded VP neurons, six recorded neurons (20.7%) from the high NaCl diet group and seven recorded neurons (30.4%) in the control group showed a decrease (>2.5 pA) in the steady state inward current held at −80 mV in response to the application of benzamil (Fig. 3 *A*). The benzamil sensitive current was obtained from the difference in the steady state currents before and after the application of benzamil. The mean benzamil sensitive current was significantly larger in VP neurons from the high NaCl group (19.1 + 4.5 nA) than from control diet group (5.2 + 1.5 nA; Fig. 3 *B*). However, the larger magnitude current may be due to the hypertrophy of VP neurons in response to the high dietary salt intake, similar to the hypertrophy observed in magnocellular neurons during dehydration (Tweedle & Hatton, 1977; Zhang *et al*. 2007). To normalize the cell size difference among VP neurons, whole cell membrane capacitance was measured. The mean whole cell membrane capacitance was significantly larger in VP neurons from the high NaCl group (37.1 + 2.9 pF) than from the control group (30.8 + 1.9 pF), indicating that a hypertrophy of VP neurons also occurred in response to the high dietary salt intake (Fig. 3 *C*). Subsequently, the benzamil sensitive current was normalized by the whole cell capacitance, and the current density (pA pF^−1^) was obtained. The mean current density was still significantly greater in VP neurons from rats fed a high NaCl diet (0.53 + 0.14 nA pF^−1^) than those fed a control diet (0.17 + 0.11 nA pF^−1^; Fig. 3 *D*). In addition, the current–voltage (*I*–*V*) relation was assessed by the voltage ramp method. A sequence of ramps (−90 to 0 mV in 5 s) was applied in the absence and presence of benzamil during current recordings (Fig. 3 *E*). The *I*–*V* relation of the benzamil sensitive current was obtained by mathematical subtraction of the current in the absence of benzamil from that in the presence of benzamil (Fig. 3 *F*). The *I*–*V* relationships revealed that the benzamil sensitive current gradually decreased at more depolarizing potentials and reached minimum at approximately −40 mV.

### Changes in the effect of benzamil on the membrane potential and firing activities

Current clamp recordings were obtained from a total of 84 VP neurons (43 neurons from a high NaCl diet and 41 neurons from the control group. Of 84 recorded neurons, 50 neurons responded to benzamil ((29 neurons (67.4%) from the high NaCl diet group and 21 neurons (51.2%) from the control diet group). The neurons that responded to benzamil were subjects for further analysis for membrane potential and firing activity. Furthermore, these neurons were classified into ‘silent’, ‘irregular firing’ or ‘phasic bursting’ neurons based on their firing pattern exhibited when no current was applied.

**Figure 4:**
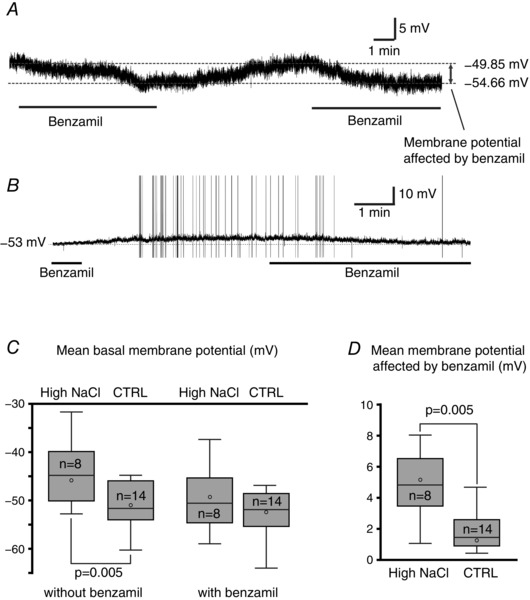
Effect of dietary NaCl on the membrane potential *A*, an example of the effect of benzamil on the basal membrane potential in VP neurons exhibiting no firing activity when no current was applied. Bath application of benzamil caused a hyperpolarization. The benzamil effective membrane potential was obtained from the difference between the membrane potential in media with and without benzamil. *B*, in some cases, non‐firing VP neurons became irregular firing neurons upon the washing off of benzamil and became non‐firing neuron once again in the presence of benzamil. *C*, the mean basal membrane potential of VP neurons from the high NaCl diet group was significantly depolarized compared to those from controls; however, the application of benzamil diminished the difference between the groups. *D*, the mean benzamil effective membrane potential was significantly higher in VP neurons from rats fed the high NaCl diet compared to those in the control group.

Twenty‐three VP neurons recorded (14 neurons from the high NaCl group and 9 neurons from the control group) were silent when no current was applied. In the silent neurons, the bath application of 2 μm benzamil caused a hyperpolarization, while washing off of the benzamil caused a depolarization close to the membrane potential before the application of benzamil (Fig. 4 *A*). In some cases, the removal of benzamil from the bath caused a membrane depolarization and subsequent firing that was blocked by the reapplication of benzamil (Fig. 4 *B*). The mean basal membrane potential was significantly (*P *< 0.005) depolarized in VP neurons within the high NaCl diet group (−46.12 + 1.9 mV) compared to the control diet group (−51.07 + 1.69 mV); however, the application of benzamil diminished the difference between the basal membrane potential between the high NaCl (−49.43 + 1.86 mV) and control groups (−52.73 + 1.76 mV; Fig. 4 *C*). The mean difference in the membrane potential in the presence and absence of benzamil was significantly greater in the high NaCl diet group (4.64 + 0.5 mV) than in the control diet group (1.62 + 0.67 mV; Fig. 4 *D*).

**Figure 5:**
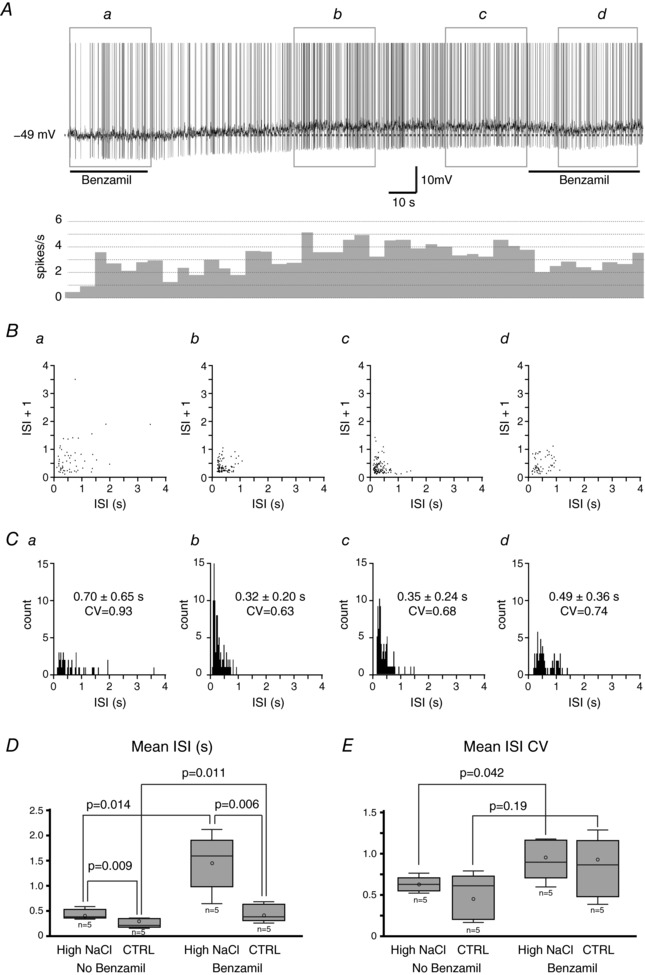
Effect of dietary NaCl on irregular firing pattern *A*, an example of a current clamp recording of a VP neuron from the high NaCl group exhibiting an irregular firing and its rate metre (10 s binned) of the firing activity when no current was applied. The washing of benzamil from the neurons caused an increase in firing frequency, while re‐application of benzamil caused a decrease. Sections *a*–*d* indicate the 30 s time frame where the interspike interval (ISI) joint interval histograms and ISI histograms were obtained in *B* and *C*, respectively. *B*, ISI joint interval histogram. Note the more dispersed dots in the presence of benzamil (*a* and *d*) compared to those in the absence of benzamil (*b* and *c*), representing a more random distribution of ISIs in the presence of benzamil. *C*, ISI histograms. The mean ISI + standard deviation along with the coefficient of variation (CV) are indicated in each ISI histogram. Note that the CV is smaller in the absence of benzamil (*b* and *c*), indicating less interspike variability. *D*, the mean ISI did not differ between groups in the absence of benzamil; however, it was significantly longer in neurons from the high NaCl diet group compared to those in the control diet group. *E*, there was no difference in the CV between groups regardless of the presence of benzamil. The application of benzamil caused a significant increase in the CV in the control diet group. However, the increase in the CV observed in neurons from the high NaCl diet group in response to benzamil was not statistically significant.

Ten VP neurons recorded (5 neurons from the high NaCl group and 5 neurons from the control group) were classified as ‘irregular firing’ neurons. An example of the irregular firing activity of a VP neuron from the high NaCl diet group, the interspike interval (ISI) joint interval histograms, and the ISI histograms are shown in Fig. 5 *A*–*C*, respectively. The firing frequency of the irregular firing VP neurons ranged from 1 to 6 Hz. In the control diet group, the application of benzamil caused a slight but significant increase in the mean ISI of the irregular firing neurons (no benzamil: 0.26 + 0.09 s; benzamil: 0.44 + 0.08 s; Fig. 5 *D*); however; the apparent increase in the mean ISI coefficient of variation (ISI CV) observed was not statistically significant (no benzamil: 0.49 + 0.12; benzamil: 0.82 + 0.16; Fig. 5 *E*), indicating that the interspike variability was not affected by benzamil. The application of benzamil also caused a significant increase in the mean ISI of irregular firing neurons in the high NaCl diet group (no benzamil: 0.44 + 0.04 s; benzamil: 1.47 + 0.55 s; Fig. 5 *D*). In contrast to the control group, the application of benzamil caused a significant increase in the mean ISI CV (no benzamil: 0.62 + 0.04; benzamil: 0.92 + 0.11; Fig. 5 *E*), indicating increased interspike variability in the presence of benzamil in the high NaCl diet group. The effect of dietary NaCl on the irregular firing activity was also analysed. The high dietary salt intake caused significant increase in the mean ISI in irregular firing neurons in both the presence of benzamil (control: 0.26 + 0.04 s; 8% NaCl: 0.44 + 0.04 s; Fig. 5 *D*) and in the absence of benzamil (control: 0.44 + 0.08 s; 8% NaCl: 1.47 + 0.55 s; Fig. 5 *D*). No difference in the ISI CV between groups was found in the presence of benzamil (control: 0.82 + 0.16; 8% NaCl: 0.92 + 0.11; Fig. 5 *E*) or the absence of benzamil (control: 0.49 + 0.12; 8% NaCl: 0.62 + 0.04; Fig. 5 *E*).

**Figure 6:**
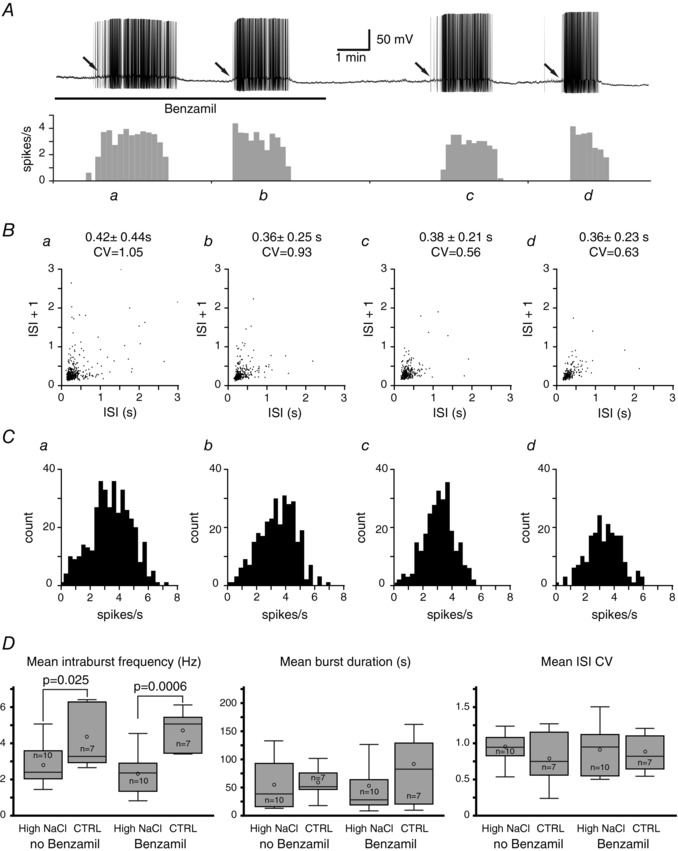
Effect of dietary NaCl on phasic bursting activity *A*, an example of current clamp recording of a VP neuron exhibiting phasic bursting activity and its rate metre (10 s binned) of the firing activity when no current was applied. Two periods of burst (*a* and *b*) occurred in the presence of benzamil, whereas two other periods of burst (*c* and *d*) occurred in the absence of benzamil. *B*, ISI joint interval histogram along with the mean ISI + standard deviation and the coefficient of variation (CV) for each bursting period indicated as *a*–*d* in *A*. *C*, intraburst frequency histogram for each bursting period. *D*, the mean intraburst frequency, burst duration, and ISI CV were not affected significantly by the application of benzamil in VP neurons from animals that were fed a high NaCl diet, compared to that from the control diet group. The mean intraburst frequency was significantly slower among VP neurons from the high NaCl diet group; however, no significant differences occurred in the mean burst duration and ISI CV between the high NaCl and control diet groups.

Seventeen VP neurons (10 neurons from the high NaCl group and 7 neurons from the control group) were characterized as phasic bursting neurons. The phasic bursting activity in VP neurons is characterized by alternating periods of firing and silence lasting tens of seconds each. An example of the phasic bursting activity of a VP neuron from the high NaCl diet group, the ISI joint interval histograms, and the ISI histograms are shown in Fig. 6 *A*–*C*, respectively. Each bursting was always preceded by slow depolarization that initiated the bursting in all phasic bursting VP neurons recorded (arrows in Fig. 6 *A*). This pre‐bursting depolarization was observed in the presence and absence of benzamil. The occurrence of bursting was certainly not affected by benzamil as bursting was observed in the presence of benzamil in every phasic bursting neuron. The mean intraburst frequency, burst duration, and ISI CV were not affected by the application of benzamil in the phasic bursting VP neurons regardless of the dietary salt intake. The effect of dietary NaCl on the phasic bursting was also analysed. The high dietary salt intake resulted in significantly slower mean intraburst frequency in the phasic bursting neurons in the absence of benzamil (control: 4.27 + 0.62 Hz; high NaCl: 2.80 + 0.37 Hz) and in the presence of benzamil (control: 4.71 + 0.44 Hz; high NaCl: 2.27 + 0.34 Hz); however, the mean burst duration (no benzamil, control: 58.9 + 10.2 s; no benzamil, high NaCl: 54.9 + 14.1 s; benzamil, control: 80.6 + 24.3 s; benzamil, high NaCl: 52.5 + 17.6 s) and ISI CV (no benzamil, control: 0.78 + 0.14; no benzamil, high NaCl: 0.93 + 0.07; benzamil, control: 0.86 + 0.10; benzamil, high NaCl: 0.89 + 0.10) were not affected by the dietary salt intake regardless of the presence of benzamil.

## Discussion

The present study demonstrated for the first time that the expression and activity of ENaC in VP neurons in the SON are influenced by dietary NaCl intake. The high dietary NaCl intake for 7 days caused a significant increase in the expressions of β and γENaC subunits in the SON and the translocation of αENaC towards the plasma membrane of magnocellular neurosecretory cells. These changes in ENaC subunit expression and subcellular distribution were associated with an increase in the benzamil sensitive current in VP neurons. In addition, the more depolarized basal membrane potentials in VP neurons in response to a high dietary NaCl intake was, at least partly, mediated by the benzamil sensitive property. The application of benzamil affected the irregular firing activity of VP neurons; however, the application of benzamil had no effect on the phasic bursting activity as neither the mean intraburst frequency, burst duration, nor interspike interval variability was affected.

### Dietary salt intake affects the expression of β and γENaC subunits, but not αENaC

This study found that high dietary NaCl intake caused a significant increase in the expression of β‐ and γENaC in the SON. One millimetre diameter tissue punches containing the entire SON were collected for qPCR. Thus, the samples also included glial and vascular cells and possibly neurons from the perinuclear zone of the SON. Our previous study found that β and γENaC immunoreactivity were exclusively located in VP‐immunoreactive neurons but not in oxytocin‐immunoreactive neurons or any other cell types in the SON (Teruyama *et al*. 2012). Consistent with our previous study, β and γENaC immunoreactivity was not found in glial and vascular cells in the present study (Fig. 2). Therefore, the increased expressions of β and γENaC in response to high dietary NaCl intake probably occurred in VP neurons in the SON.

ENaC expression in the epithelia is mainly regulated by the primary mineralocorticoid aldosterone through the mineralocorticoid receptor (MR) (Garty & Palmer, 1997). Our previous study found that MR and ENaC are colocalized in VP neurons in the SON and PVN (Teruyama *et al*. 2012; Haque *et al*. 2015), suggesting that ENaCs in VP neurons are also regulated by aldosterone. Intriguingly, the present study found that a NaCl deficient diet, which is known to cause an increase in aldosterone in the general circulation (Frindt *et al*. 1990; Pacha *et al*. 1993; Asher *et al*. 1996), did not cause a change in ENaC expression in the SON, although it caused an increase in αENaC mRNA in the kidney, consistent with findings from previous studies (Renard *et al*. 1995; Asher *et al*. 1996; Escoubet *et al*. 1997; Stokes & Sigmund, 1998; Masilamani *et al*. 1999; MacDonald *et al*. 2000). These findings suggest that elevated aldosterone in the general circulation has no effect on ENaC expression in VP neurons. Despite the lipophilic nature of steroids, aldosterone has a surprisingly poor penetration of the blood–brain barrier (reviewed in Geerling & Loewy, 2009), which may explain the absence of a change in ENaC expression in VP neurons in response to plasma aldosterone. Accumulating evidence now indicates that aldosterone is synthesized in the brain. In rats, the transcripts of aldosterone synthase (CYP11B2) was detected in various brain regions including the hypothalamus (MacKenzie *et al*. 2000). The capability of brain tissue to synthesize aldosterone was also demonstrated *in vitro* (Gomez‐Sanchez *et al*. 2005). Recently, *in situ* hybridization demonstrated the presence of aldosterone synthase in magnocellular neurons in the SON (Wang *et al*. 2016). Moreover, hypothalamic aldosterone production appears to be stimulated by a high concentration of Na^+^ in CSF. In rats, intracerebroventricular (ICV) infusion of Na^+^‐rich ACSF caused an increase in hypothalamic aldosterone concentration and blood pressure, and both were prevented by ICV infusion of an aldosterone synthase inhibitor (Huang *et al*. 2008). These findings suggest that the high salt diet induced expression of β‐ and γENaC in the SON found in the present study were mediated by aldosterone that was synthesized locally in response to a high dietary salt intake.

Presumably ENaC in VP neurons is also regulated by aldosterone, which suggests that aldosterone induces expression of different ENaC subunits according to cell types and/or organs. Recently, chronic subcutaneous aldosterone infusion for 10 days was found to increase γENaC mRNA level in the subfornical organ (SFO) of the hypothalamus in rats that were provided with saline drinking fluid (aldosterone salt induced hypertension model) (Wang *et al*. 2016). Although the SFO is a structure in the brain, it is known that it lacks the blood–brain barrier and shows high expression of MR (Amin *et al*. 2005). Therefore, aldosterone from subcutaneous infusion may directly affect the expression of γENaC in the SFO. Aldosterone induced γENaC expression was also found in taste receptor cells (Lin *et al*. 1999) where ENaC plays a critical role in transducing salt taste (Gilbertson & Kinnamon, 1996; Kinnamon & Margolskee, 1996; Lindemann, 1996). Increases in blood aldosterone enhanced the intensity of the apical immunoreactivity for βENaC and γENaC, and increased the amplitude of amiloride sensitive current in taste cells (Lin *et al*. 1999), indicating an increased salt appetite in response to aldosterone. These findings further support the idea that aldosterone induced βENaC and γENaC expression in VP neurons.

Because the high dietary salt intake‐induced expression of βENaC and γENaC was associated with enhanced ENaC activity in VP neurons, increased expression of βENaC and γENaC is likely to be sufficient to enhance ENaC activity without an increase in αENaC expression. A similar observation was made in cultured collecting duct cells; increased expression of the αENaC and/or βENaC by a regulated adenovirus system did not alter Na^+^ transport, while a small increase in γENaC expression alone increased Na^+^ transport approximately threefold (Husted *et al*. 2007). Thus, the expression of γENaC may be the rate‐limiting factor in ENaC activation. It is also known that proteolytic cleavage of γENaC to release an extracellular inhibitory domain (Passero *et al*. 2010) is critical for full channel activation (Berman *et al*. 2015; Shobair *et al*. 2016). Intriguingly, salt deprivation enhanced the proteolytic cleavage of the γENaC subunit in the kidney collecting tubule (Masilamani *et al*. 1999), implying that similar cleavage of the γENaC subunit may result in response to changes in dietary salt intake. Lastly, there is a possibility that the ENaC subunit composition is altered in response to changes in βENaC and γENaC expressions. The three ENaC subunits assemble into a heteromultimer to form a functional complex (Berdiev *et al*. 1998; Firsov *et al*. 1998; Snyder *et al*. 1998); however, the stoichiometry of ENaC remains uncertain and controversial since reports of the total number of subunits vary from four to nine (Firsov *et al*. 1998; Kosari *et al*. 1998; Snyder *et al*. 1998; Eskandari *et al*. 1999; Staruschenko *et al*. 2004, 2005). One of the possible reasons for the inconsistency in determining ENaC subunit composition may be an alteration in the composition of the subunits. Single channel properties of ENaCs in native tissues vary considerably, and alteration of subunit composition is suggested as a cause for this variation (Fyfe & Canessa, 1998). Nevertheless, changes in ENaC subunit expression in response to dietary salt intake suggest that subunit composition may be altered, which may be physiologically important.

### Dietary salt intake affects the subcellular distribution of ENaCs

Because ENaC is a transmembrane ion channel that becomes functional once it is inserted in the cell membrane, the translocation of ENaC into or near the cell membrane may be observed in neurons that possess the abundant active form of ENaC. The present study revealed that high dietary NaCl intake caused a redistribution of αENaC immunoreactivity towards the plasma membrane. Because it is generally agreed that the full activity of ENaC requires all three subunits (Canessa *et al*. 1994; McDonald *et al*. 1994; Snyder, 2000), αENaC immunoreactivity observed near the cell membrane may represent a fully assembled functional ENaC. Similar to our observation, ENaC subunits were displayed in the sub‐apical cytoplasm and in the apical membrane of the collecting duct principal cells in rats (Masilamani *et al*. 1999) and mice (Loffing *et al*. 2000) on a low NaCl diet. Because VP induces translocation of intracellular pools of pre‐existing ENaC subunits to the apical membrane in the cortical collecting duct cells (Schafer & Hawk, 1992), the increased sub‐membrane distribution of ENaC observed may be due to increased VP release within the SON occurs in response to an osmotic challenge (Ludwig *et al*. 2005). This finding implies that high dietary NaCl intake activates ENaC insertion into the cell membrane.

### Not all VP neurons respond to the application of benzamil

In the present voltage clamp experiment, only a minority of VP neurons showed a clear decrease in the steady state inward current in response to benzamil (approximately 30% in control and 20% in high salt diet group). The exact cause of the poor percentage of VP neurons that responded to benzamil in voltage clamp configuration is unknown. One possible explanation is that the relatively small effect of benzamil on the current was not detected, because an opposing gradual increase in the leak current that often occurred in our recordings cancelled out the benzamil induced decrease in the leak current. In fact, a considerably larger fraction of VP neurons responded to benzamil in the current clamp experiments (approximately 50% in control and 70% in high salt diet group). Another possibility is that the ENaCs are not in an active form in *in vitro* slice preparations and only basal activity can be recorded. For instance, the extracellular proteolytic enzymes that are required for proteolytic activation of ENaC (Berman *et al*. 2015; Shobair *et al*. 2016) may not be available *in vitro*. Nonetheless, it seems that not all VP neurons respond to benzamil. The lack of response to benzamil from some VP neurons is consistent with our previous study that found that not all VP immunoreactive neurons had β and γENaC immunoreactivity, while αENaC immunoreactivity was found essentially in all VP immunoreactive neurons (Teruyama *et al*. 2012). Immunocytochemistry in the present study also found that only a handful of magnocellular neurons were clearly defined by βENaC immunoreactivity, and intense γENaC immunoreactivity was observed only in a fraction of the magnocellular neurons in each section of the SON. Collectively, these findings suggest that not all VP neurons express or possess functional ENaCs. A more important query is whether ENaC activity in a potentially small population of VP neurons exerts any significant physiological role. It is now known that dynorphin, an endogenous opioid peptide, is located in the same neurosecretory vesicles as VP, and both dynorphin and VP are released from the soma and dendrites to cause activity dependent modulation of VP neuron activity via kappa‐opioid receptors and VP receptors, respectively (Brown *et al*. 2007). Therefore, ENaC activity in a small population of VP neurons may affect the entire population of VP neurons within the SON.

### Dietary salt intake affects membrane potential of VP neurons via modulation of ENaC

The present study also demonstrated that some of the changes in the electrophysiological properties of VP neurons in response to high dietary salt intake are due to the modification in ENaC activity. First, voltage clamp experiments showed that the current density of benzamil sensitive inward leak currents was specifically enhanced in response to high dietary salt intake. Second, current clamp experiments showed that the benzamil sensitive property is responsible for the more depolarized basal membrane potential of VP neurons in the high salt diet group. These findings suggest that ENaC is at least partly involved in modification of the resting membrane potential appropriate for a particular physiological state.

The repetitive firing of VP neurons is critically modulated by intrinsic membrane properties, the Ca^2+^ dependent depolarizing after potentials (DAPs) (Teruyama & Armstrong, 2007; Armstrong *et al*. 2010). When spikes occur close enough together, DAPs generate a persistent plateau potential that sustains continuous firing (Andrew & Dudek, 1983; Brown & Bourque, 2006); however, the DAP is also a voltage‐dependent potential (Andrew & Dudek, 1984) and is evoked only when the basal membrane potential is close to the threshold of activation of action potentials. In our recording condition, the firing in VP neurons was observed only within a narrow range of the membrane potential (−48 mV to −55 mV). Slightly more depolarized potentials resulted in phasic bursting or continuous firing, whereas a slightly hyperpolarized potential resulted in repetitive firing or no firing at all. These results indicate that small alterations in the resting membrane potential by ENaCs affect how synaptic potentials summate to cross the threshold for spike generation.

### ENaC activity does not affect the phasic bursting of VP neurons

The phasic bursting activity of VP neurons is thought to be the most efficient firing pattern for the secretion of VP from the neurohypophysis (Bicknell & Leng, 1981; Brown *et al*. 2007, 2008). The potential effect of ENaCs on the phasic bursting activity is, therefore, of great interest to understand the role of ENaC in the release of VP. Despite the significant role of ENaCs in the modulation of the resting membrane potential, ENaCs appear to have no role in the regulation of the phasic bursting activity in the absence of synaptic inputs, as neither the intraburst frequency, burst duration, nor ISI CV were affected by the application of benzamil. One of the reasons for this may be due to the fact that the phasic bursting neurons were considerably more depolarized and, therefore, the driving force for Na^+^ was diminished. Indeed, the application of benzamil caused a significant increase in the mean ISI in the irregular firing neurons where the membrane potentials were slightly more hyperpolarized than those of the phasic bursting neurons.

In addition, phasic bursting is strongly regulated by spike‐afterpotentials, such as DAP and afterhyperpolarizations (AHPs) (Stern & Armstrong, 1996; Teruyama & Armstrong, 2002, 2005). When sufficient summation of DAPs induces a plateau potential that gives rise to the bursts (Andrew & Dudek, 1983, 1984; Andrew, 1987; Brown & Bourque, 2006), the train of action potentials in turn elicits the AHPs in an activity dependent manner (Kirkpatrick & Bourque, 1996) that results in a suppression of the firing rate. The present findings infer that ENaC has little influence on phasic bursting because it is primarily regulated by spike‐afterpotentials once started.

The potential role of ENaCs in the initiation of phasic bursting was also assessed. Phasic bursting *in vivo* is initiated by synaptic inputs (Brown & Bourque, 2006); however, phasic bursting occurred *in vitro* brain slices in which VP neurons were exposed to DNQX and picrotoxin to block all the synaptic inputs in our recording condition and in others (Li *et al*. 2007). Therefore, the initiation, maintenance, and termination of bursting were entirely the result of intrinsic membrane properties *in vitro*. The phasic bursting of VP neurons was always preceded by a slow depolarization that led to the bursting. Presently, the mechanism underlying this slow depolarization is unknown; however, the fact that the slow depolarization that led to the phasic bursting occurred in the presence of benzamil alone provides sufficient evidence that ENaC is not involved in the initiation of phasic bursting, at least, *in vitro*.

### Dietary salt intake affects the firing activity of VP neurons

Among the phasic bursting neurons, high dietary salt intake caused a slow intraburst frequency in VP neurons. Consistent with this finding, the mean ISI of the high NaCl group among the irregular firing neurons was significantly longer than from the control group. Because these changes were observed in the presence of benzamil, other intrinsic properties that hinder the firing frequency must be involved in the slow intraburst frequency in response to a high dietary salt intake. One such property may be a medium AHP (mAHP) that is mediated by small conductance Ca^2+^ activated K^+^ ion (SK) channels (Sah & Faber, 2002). In oxytocin neurons, the amplitude of the SK current increased during lactation when physiological demand for oxytocin is high (Teruyama & Armstrong, 2005). The enhanced SK current results in a stronger suppression of the firing rate presumably to avoid secretory fatigue associated with strong activation. It is interesting whether a similar plasticity in mAHP is present in VP neurons under high physiological demand for the hormone. Nevertheless, the enhanced intrinsic membrane property may restrain the firing frequency while VP neurons are triggered to a greater excitability during chronic osmotic challenge, perhaps to prevent secretory fatigue.

The slow intraburst frequency in the high NaCl diet group is somewhat contradicted by *in vivo* recordings that showed the mean basal firing rate of VP neurons from dehydrated rats was greater than in euhydrated rats (Dyball & Pountney, 1973; Walters & Hatton, 1974; Wakerley *et al*. 1978; Scott *et al*. 2009). The increased firing rate during a burst was most probably due to an increased synaptic drive (Di & Tasker, 2004), rather than to intrinsic membrane properties. The present study was conducted in *in vitro* brain slice preparations in which synaptic inputs were pharmacologically blocked. Therefore, the findings from the present study are not comparable to those from *in vivo* studies. However, the findings from the present study suggest that ENaC activity would augment the synaptic drive by depolarizing the basal membrane potential close to the action potential threshold during hormonal demand.

### Conclusion and remarks

Results from the present study strongly suggest that high dietary salt intake causes depolarization of the basal membrane potential in VP neurons via modulation of the expression and translocation of ENaCs. The enhanced ENaC activity probably augments the synaptic drive by depolarizing the basal membrane potential close to the action potential threshold during hormonal demand in response to high dietary NaCl intake, because phasic bursting in VP neurons *in vivo* is initiated by synaptic inputs (Brown & Bourque, 2006). Therefore, modulation of ENaCs are potentially powerful means to regulate the firing activity that ultimately controls hormone secretion according to physiological demands. However, ENaC appears to have no role in the regulation of phasic bursting that is intrinsically initiated without synaptic inputs.

## Additional information

### Competing interests

The authors have no competing interests to declare.

### Author contributions

The experiments were performed at Louisiana State University in the laboratory of R.T. R.T. contributed to conception and design of the work. K.S., M.H., R.G., and R.T. contributed to the acquisition, analysis or interpretation of data for the work. Y.U. contributed to the creation of the experimental animal model. K.S. and R.T. contributed drafting the work or revising it critically for important intellectual content. All authors have approved the final version of the manuscript and agree to be accountable for all aspects of the work in ensuring the questions related to the accuracy or integrity of any part of the work are appropriately investigated and resolved. All persons designated as authors qualify for authorship, and all those who qualify for authorship are listed.

### Funding

The work was supported by NIH National Heart, Lung, and Blood Institute Grant R01 HL115208 (R.T.).
